# The vaginal microbiome of sub-Saharan African women: revealing important gaps in the era of next-generation sequencing

**DOI:** 10.7717/peerj.9684

**Published:** 2020-08-18

**Authors:** Nkechi Martina Odogwu, Oladapo O. Olayemi, Akinyinka O. Omigbodun

**Affiliations:** 1 Pan African University of Life and Earth Science Institute, Department of Obstetrics and Gynecology, University College Hospital, University of Ibadan, Ibadan, Oyo, Nigeria; 2 Department of Obstetrics and Gynecology, College of Medicine, University College Hospital, University of Ibadan, Ibadan, Oyo, Nigeria

**Keywords:** Vaginal microbiome, Sub-Saharan African women, High throughput sequencing, Next generation sequencing

## Abstract

Accurate characterization of the vaginal microbiome remains a fundamental goal of the Human Microbiome project (HMP). For over a decade, this goal has been made possible deploying high-throughput next generation sequencing technologies (NGS), which indeed has revolutionized medical research and enabled large-scale genomic studies. The 16S rRNA marker-gene survey is the most commonly explored approach for vaginal microbial community studies. With this approach, prior studies have elucidated substantial variations in the vaginal microbiome of women from different ethnicities. This review provides a comprehensive account of studies that have deployed this approach to describe the vaginal microbiota of African women in health and disease. On the basis of published data, the few studies reported from the African population are mainly in non-pregnant post pubertal women and calls for more detailed studies in pregnant and postnatal cohorts. We provide insight on the use of more sophisticated cutting-edge technologies in characterizing the vaginal microbiome. These technologies offer high-resolution detection of vaginal microbiome variations and community functional capabilities, which can shed light into several discrepancies observed in the vaginal microbiota of African women in an African population versus women of African descent in the diaspora.

## Introduction

Accurate identification of the vaginal microbiota has broadened our understanding of the aetiology of genital tract infections and adverse pregnancy outcome. Most post pubertal women have a vaginal microbiome dominated by *Lactobacilli*, which enhances vaginal community stability (De Seta et al., 2019). Over 130 *Lactobacillus* species have been reported, and 20 of these species have been isolated from the vagina (Zhou et al., 2004; Ravel et al., 2011). By hierarchical clustering analysis, Ravel et al. (2011) classified these bacteria into community state types (CST), including CST I (*L. crispatus* dominated), CST II (*L. gasseri* dominated), CST III (*L. iners* dominated), CST V (*L. jensenii* dominated), and CST IV (a heterogeneous group of strict anaerobes). A healthy vaginal community may often be dominated by one or two vagitypes (Zhou et al., 2010a; Ravel et al., 2011). A deviation from a ‘*Lactobacillus’* vaginal profile primes abnormal conditions such as bacterial vaginosis (BV) (Nelson et al., 2015), and aerobic vaginitis (AV) (Donders et al., 2002). Aerobic vaginitis describes a state of bacterial colonization by aerobic pathobiont such as Group B *Streptococcus* and *E.coli* (Donders et al., 2002), whereas BV is a condition characterized by an heterogenous mixture of Bacterial Vaginosis Associated Bacteria (BVAB) including *Bifidobacterium* spp*, Dialister* spp*, Prevotella* spp*, Atopobium* spp*, Megasphaera* spp*, Group B Streptococcus, Mycoplasma* spp*, Bacteriodes* spp*, Mobiluncus* spp*, Gardnerella* spp, *Sneathia* spp, *Finegoldia* spp, *Peptoniphilus* spp, *Anaerococcus* spp, *Corynebacterium* spp and other taxa of the order Clostridiales (Smith & Ravel, 2017). These bacteria are classified as community state type IV (Ravel et al., 2011; Gajer et al., 2012). A higher prevalence (51.4%) of a BV-associated profile has been reported in African and African-American women, double the prevalence of 23.2% found in White women (Fettweis et al., 2014). This condition predisposes to Pelvic inflammatory diseases (Ness et al., 2004), increased HIV and STI acquisition (Martin et al., 1999; Schwebke, 2003; Wiesenfeld et al., 2003; Coleman et al., 2007; Cherpes et al., 2003) and has been noted as a major risk factor for pre-term premature rupture of membranes (PPROM), pre-term births (PTB), early miscarriage and ascending urogenital infections (Hillier et al., 1995; Nelson et al., 2009). For women with a *Lactobacillus*-dominated profile, those whose vaginal profile are dominated by *L. crispatus * are less likely to develop vaginal dysbiosis whereas women with *L. iners* dominated vaginal profile are easily prone to vaginal dysbiosis (Verstraelen et al., 2009). There have been several observations regarding variations in the vaginal microbiome between women from different ethnicities. Caucasians and Asians are reportedly known to have a significant amount of *Lactobacillus* dominated vaginal profile, compared to Black women (Zhou et al., 2010a; Zhou et al., 2010b; Fettweis et al., 2014). Furthermore, Black women more often develop BV during pregnancy and becomes susceptible to preterm birth compared to European women (Paige et al., 1998; Kramer & Hogue, 2008). The basis for these ethnic differences in the vaginal microbiome composition remains unclear. With Nugent score system (a traditional method of bacterial identification), several studies have observed vaginal colonization with BVAB in African and African-American women. The Nugent score is a gram staining score criteria used to quantify bacteria of vaginal samples such that a high score depicts BV while a low score translates to a healthy vagina (Nugent, Krohn & Hillier, 1991). Prior studies had reported high vaginal Nugent scores in African-American women in contrast to women of European ancestry (Nugent, Krohn & Hillier, 1991; Royce et al., 1999; Ness et al., 2003; Fiscella & Klebanoff, 2004). However, this traditional method of bacterial identification only gives details of bacterial morphotype and not their genetic constitution, consequently leaving a sizeable fraction of the vaginal microbiota undeciphered. For easy characterization of the complex vaginal microbial communities, impenetrable by traditional culture techniques, the Human Microbiome Project (HMP) proposed the deployment of DNA sequencing technology (NIH et al., 2009; HMP, 2012). Most notable is the profiling of the 16S rRNA maker-gene. Despite some promising results obtained with deploying this method, there have been conflicting reports about the vaginal microbiota of African women. These conflicting reports stem from the differences observed between the vaginal microbiome of African women in the Western hemisphere and those in sub-Saharan Africa. This suggests that there may be a geographical influence on the vaginal microbiome and calls for more geographically-tailored community-scale studies. The objective of this review is first to provide a comprehensive account of the vaginal microbial profile in non-pregnant, pregnant and puerperal women of African ancestry in studies that have deployed 16S rRNA sequencing, provide better insight (and possibly reveal important gaps) in vaginal microbiome science and, secondly, we seek to provide insight into other refined cutting-edge technologies for the identification of vaginal bacterial communities.

## Methodology

### Search Strategy

To select eligible and relevant literature for this review, we conducted a peer-reviewed article search strategy using important key words. Searches included articles and grey literature including reviews and original research published in PubMed, PubMed central, Google Scholar, Scopus, Web of Science, Evidence-Based Medicine, Biosis preview, Biological Abstract and African Journal Online database.

### Identification of Eligible Studies

**Figure 1:**
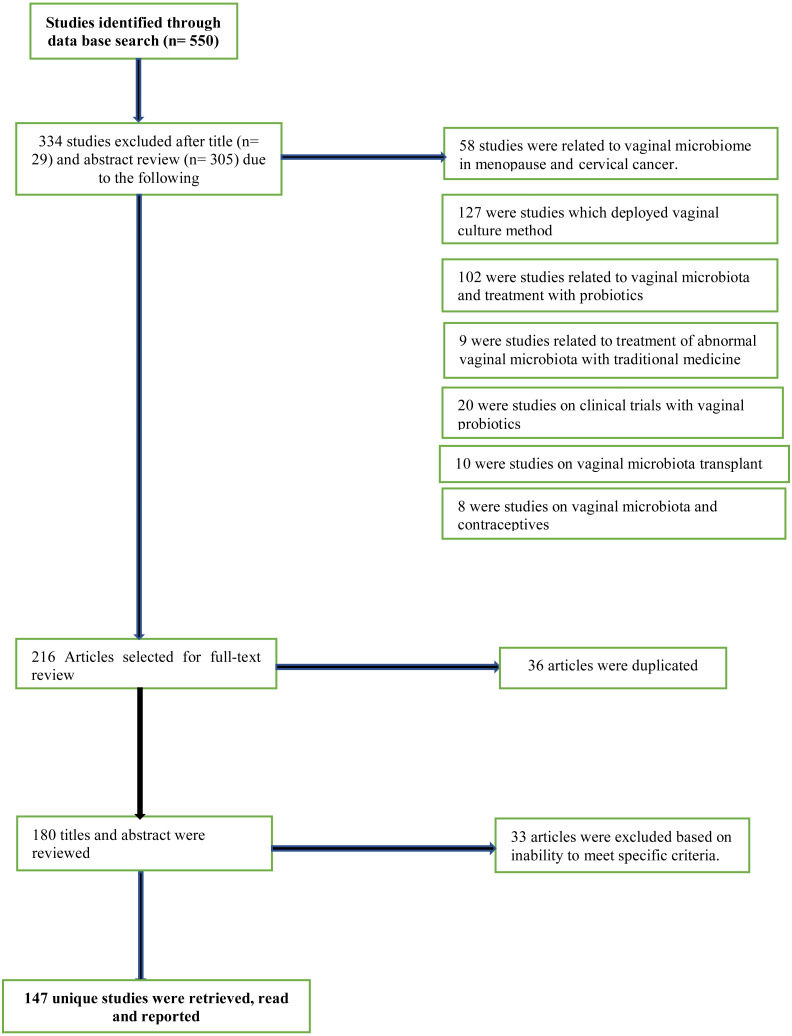
PRISMA flow diagram for data representation and analysis.

From the database search, titles, abstracts and full-text versions of articles were identified and screened for potential eligibility. After title, abstract and full-text reviews, irrelevant and non-eligible articles were screened out, leaving only potentially relevant ones. Eligible articles were studies written in English language. Multiple keywords were used for the literature search both alone as well as in combination. Some of the important keywords used for literature search were vaginal microbiome, vaginal microbiota studies, sequencing approach, amplicon marker gene sequencing, next-generation sequencing platforms, vaginal microbiota of African women, postpartum vaginal profile, vaginal microbiota during pregnancy in African cohorts. Original research and critical reviews were both included and studies irrelevant to the scope of this review were excluded described in Fig. 1. All three investigators independently reviewed titles/abstracts and full text for eligibility. The reference lists of eligible articles were also screened to detect relevant articles that were not identified by the initial search strategy.

### Evaluation of eligible studies

All investigators independently extracted data from the selected search database and downloaded article. Any discrepancies in data extraction and risk of bias assessment were resolved by consensus. All authors reviewed article, titles and abstracts independently and retrieved full articles that potentially met the inclusion criteria. Having identified the studies that met the inclusion criteria, full text versions of these articles were read and saved in personal devices.

## Results

### Selection of eligible studies

Of 550 unique titles/abstracts identified from the database search, 29 were excluded after title review and 305 after abstract review, leaving 216 eligible articles for full text review (Fig. 1). Of these, 36 articles were discarded as duplicates as they were found more than once in the selected database search engine. The remaining 180 titles and abstracts of articles were reviewed again and another 33 articles were excluded, based on their irrelevance or inability to meet specified criteria. The remaining 147 full-text studies were retrieved and read in full (Fig. 1).

### The vaginal ecosystem

The vaginal epithelium comprises of three cell layers, superficial, intermediate, and basal. The epithelial mucosa of the lower genital tract is extensively populated by commensal microorganisms, while the tissues of the upper genital tract are not colonized by commensals thus are less prone to infection (Rampersaud, Randis & Ratner, 2012). The vaginal microbiome is distinctive for its relatively simple biodiversity, low species richness and numerous *Lactobacillus* species Human Microbiome Project Consortium (2012). *Lactobacillus* stand out as key players in modulating reproductive health in post pubertal women by exerting a protective effect on the vagina. The mechanisms by which *Lactobacilli* modulate reproductive health of women is not known with certitude but may be by it acting as a competitor with other pathogenic organisms for nutrients, epithelial cell receptors and space (Boris et al., 1998). Other putative mechanisms are the release of metabolites and secretion of bacteriocins to maintain a low hostile vaginal pH (Martin & Suarez, 2010) and the production of lactic acid which protects the vagina from colonization by other species (Witkin et al., 2013).

A vaginal microenvironment less dominated by *Lactobacilli* predisposes to adverse clinical conditions like BV (Allsworth & Peipert, 2007; Srinivasan & Fredricks, 2008) or non-specific vaginitis (Amsel et al., 1983). Due to the multifaceted function of the vagina, coupled with its anatomical location, it may be influenced by hormones, menstruation, douching practices, contraceptives, sexual intercourse and the gastrointestinal microflora from the rectum (Reid, 2018). Several studies have reported a *Lactobaccillus* depleted vaginal profile in African-American and Hispanic women (Shendure, Porreca & Reppas, 2005; Ravel et al., 2011; Zhou et al., 2010b). Jespers et al. (2014) noted similar findings in a cohort of African women. Interestingly, others have also reported a high prevalence of BV in sub-Saharan African women (Gautam et al., 2015; Torrone et al., 2018). With the advent of next generation sequencing, explicit identification of vaginal microbiota has been made possible. This move has also provided a platform for scientists to make comparisons across populations and construct novel research questions in vaginal microbiology and ecology.

### Next generation sequencing

NGS describes a method of sequencing where millions of oligonucleotides sequencing fragments are executed in parallel, giving rise to large number of sequencing outputs (Mendz, Kaakoush & Quinlivan, 2016). Until the HMP was established, therapeutic interventions and treatment of vaginal disorders has been unsuccessful because the identification of the complex vaginal microbial community depended on the tripods of clinical diagnosis, microscopy and basic culture technique (White et al., 2011). DNA Sequencing was first elaborated by Sanger in a method known as Sanger sequencing or the chain-terminator methods (Sanger, Nicklen & Coulson, 1977). It was developed in 1977 and remains the “gold standard” in molecular diagnostics. It operates by utilizing DNA polymerase to generate a complementary copy to a single stranded DNA template which bind to a given primer. Due to primer binding, the preceding bases of the sequences produced are usually of poor quality (Sanger, Nicklen & Coulson, 1977; Adams, 2008). In addition, it is time consuming and expensive. With these limitations, Sanger sequencing has been replaced by other powerful next-generation sequencing methods which has improved the identification of the myriads of microbes even in larger scale (NIH et al., 2009; Human Microbiome Project Consortium, 2012). A major application of NGS is in phylogenetic sequencing analysis.

### Application of next generation sequencing

#### Phylogenetic marker gene (16SrRNA gene) sequencing

The 16S rRNA gene was first described by Carl Woese and George Fox (Woese, Kandler & Wheelis, 1990; Woese & Fox, 1977) and was later explored for phylogenetic analysis (Lane et al., 1985). Overtime, the 16S rRNA gene has been tagged a reliable molecular clock revealing sequences from distantly related bacterial lineages (Tsukuda, Kitahara & Miyazaki, 2017). It has been widely used in characterization of vaginal microbial communities in several cohort (Aagaard et al., 2012; Gajer et al., 2012; Huang et al., 2014; Walther-António et al., 2014; Fettweis et al., 2019; Ceccarani et al., 2019). The 16S rRNA gene has a length of approximately 1,500 bp which is sufficient for bioinformatics analysis (Janda & Abbot, 2007). Bacterial 16S rRNA genes generally comprises of nine “hypervariable regions” that demonstrate considerable sequence diversity among various bacterial species and can be used for species identification (Van de Peer, Chapelle & De Wachter, 1996; Chakravorty et al., 2007). Of these 9 variable regions, VI-V3, V4, and V4-V5 offers a genus level sequence resolution (Kim, Morrison & Yu, 2011). The degree of conservation widely varies between hypervariable regions. More conserved regions are associated with high taxonomic level while a less conserved regions with a lower taxonomic level (Yang, Wang & Qian, 2016). It is best to choose two hypervariable regions to identify bacteria because no single hypervariable region is able to distinguish among all bacteria. Making such a choice increases the advantage of employing 16S rRNA gene analysis for bacterial identification (Tao et al., 2017).

### Protocol for 16S rRNA gene sequencing

16S gene sequencing has shown its efficacy in both deciphering bacterial species in environmental specimen and establishing phylogenetic relationship between them (Shah et al., 2011; Eren, Ferris & Taylor, 2011). This analysis has a robust but simplified protocol given that it requires only polymerase chain reaction (PCR) and sequencing. First, an amplicon of the 16S gene is obtained through PCR. Amplicons are then sequenced by targeting the hypervariable regions of choice. The sequence obtained can be matched with a reference sequence from an existing DNA database. These signature nucleotides (reference sequences of 16S rRNA gene) allows for taxonomical classification and identification by basis of similarities to already known sequences in preceding databases (Chanama, 1999; Barghoutti, 2011; Mizrahi-Man, Davenport & Gilad, 2013). Furthermore, several bioinformatic pipeline can be used to analyze the resulting sequences including QIIME 2 (Bolyen et al., 2019), MOTHUR, USEARCH-UPARSE (for OTU-level), DADA2, USEARCH-UNOISE3(for ASV-level) (Prodan et al., 2020). Existing NGS platforms for 16S rRNA sequencing are described in Table 1.

**Table 1:** Next Generation Sequencing platforms.

Sequencing method	Sequencing system	Detection/Principle	Length	Advantage	Disadvantage
Pyrosequencing	Roche/454 GS FLX Titanium and the GS Junior sequencer	Optical detection, Uses DNA polymerase to synthesize complementary strands to a single stranded template Provides only one type of deoxynucleotide triphosphate base in a single cycle of the reaction. (Shendure, Porreca & Reppas, 2005; Mardis, 2013; Goodwin, McPherson & McCombie, 2016).	0.4–1 Kb Give rise to shorter fragments. Usually produce approximately 400 bp reads (Schuster, 2007)	Long read length of 400–1,000 nucleotides compared to sanger sequencing (Shendure, Porreca & Reppas, 2005; Mardis, 2013; Goodwin, McPherson & McCombie, 2016; Roche, 2020). Maximum throughput performance approximately 700 Mb (Roche, 2020)	High cost. Challenging sample preparation High error prone rate especially within homopolymers regions (Claesson et al., 2009; Loman et al. (2012).
Ion semiconductor-based sequencing	Ion PGM/Ion Torrent (Thermofischer, 2020)	Utilizes the release of H+ during sequencing to detect the sequences of clusters (ABM, 2020; Thermofischer, 2020)	Read length of approximately 100 to 200 nucleotide bp (Thermofischer, 2020)	More cost effective, time efficient and versatile (Thermofischer, 2020) It has a very low error rate of 1%, thus accuracy is guaranteed (Thermofischer, 2020)	Lower throughput of 10 Mb to 15 Gb compared to illumina) Produces indel error (Thermofischer, 2020)
Sequencing by synthesis (SBS) using a reversible terminator chemistry approach or cyclic reversible terminator (CRT) based sequencing	Illumina Genome Analyzer II/IIX, Illumina MiniSeq, MiSeq, NextSeq, HiSeq and HiSeq X (Illumina, 2020)	Requires step by step incorporation of reversible florescent and terminated nucleotides for DNA sequencing (Rodrigue et al., 2010; Goodwin, McPherson & McCombie, 2016; ABM, 2020). Florescence/optical detection (Bentley et al., 2008; Illumina, 2020), Overcomes the disadvantages of pyrosequencing by only incorporating a single nucleotide at a time thus reducing error prone rate with homopolymers regions (Mardis, 2013; Buermans & Den Dunnen, 2014; ABM, 2020). Associated with high error rate with increased read lengths (Bentley et al., 2008; Illumina, 2020)	Read length ranges from 150 to 300 bp (Goodwin, McPherson & McCombie, 2016; Illumina, 2020) Give rise to shorter fragments (illumina MiSeq 400–700 bp reads) (Schuster, 2007; Shendure, Porreca & Reppas, 2005; Mardis, 2013; Goodwin, McPherson & McCombie, 2016; Rodrigue et al., 2010)	Very high-through put (Harismendy et al., 2009; Illumina, 2020) Up to 99.5% accuracy is guaranteed (Bentley et al., 2008; Illumina, 2020) Less prone to homopolymer error	Long run time (Illumina, 2020)
Sequencing by ligation	SOLiD	Florescence/optical detection, Uses DNA ligase for sequence extension Does not utilize a DNA polymerase to incorporate nucleotide instead relies on 16 8mer oligonucleotide probes labelled by four different florescent dyes (Hoppman-Chaney et al., 2010; Thermofischer, 2020) Requires 5 sequencing primer for the entire reaction (Hoppman-Chaney et al., 2010; ABM, 2020; Thermofischer, 2020)	Produces 25–75 bp, 1 × 75 or 2 × 60 bp (Goodwin, McPherson & McCombie, 2016)	Very high-throughput (Thermofischer, 2020)	Give rise to shorter fragments/short read length (Thermofischer, 2020)
Single-molecule real-time sequencing (SMRT)	Pacific Biosciences (Pacb, 2020)	Optical detection Requires the addition of labelled phospho-linked nucleotides unto immobilized DNA template and polymerase. This incorporation is detected by specific fluorescent light emission which continually generate high throughput sequence reads (Pacb, 2020)	Give rise to approximately 20,000 bp to 10 Gb read length (Eid et al., 2009; Carneiro et al., 2012; Pacb, 2020)	Millions of sequence reads are produced (Pacb, 2020)	Prone to error due to million reads generated and wrong interpretation of nucleotide (Eid et al., 2009; Carneiro et al., 2012; Pacb, 2020)
Nano pore-based principle	Oxford Nanopore technologies (GridION X5 and PromethION)	DNA is sequenced directly by measuring the change in current flow due to the passage of molecule through a nanopore embedded within a membrane (Jain et al., 2016; Loose, Malla & Stout, 2016) Requires the use of sensors to detect changes in Ionic current (Nanopore tech, 2020)	Read length is approximately 1 Mb (Nanopore tech, 2020)	Has a base calling accuracy of 99% (Nanopore tech, 2020)	Requires expertise for reproducibility. Prone to large indel error Homopolymers cannot be accurately sequenced since it is difficult to differentiate the nanopore signals due to similar type of “leaving” and “entering” nucleotide (Goodwin, McPherson & McCombie, 2016)
Optical mapping principle	Bionano technologies	Based on the possibility to fluorescently label sequence-specific traits of long, high-molecular weight DNA (up to 1 Mb) to have an optical barcode per each DNA molecule. DNA is then loaded in nanotunnels and channels where it is linearized and imaged by a high-resolution camera. The images are then converted into digital label patterns (Bionanogenomics, 2020)	Larger read produced compared to other NGS	High detection capacity (Bionanogenomics, 2020)	Requires expertise for reproducibility.

### Vaginal microbiota in non-pregnant African women

The vaginal microbial communities have been studied in multiple levels, from morphological descriptions to understanding the genetic signature of microbes and how the mixtures of microbes could promote or disrupt reproductive outcome. By microscopy, the vaginal microbiota of Black women are reported to correlate with high Nugent Scores and a low proportion of *Lactobacilli* compared to their European counterparts (Nugent, Krohn & Hillier, 1991; Royce et al., 1999; Ness et al., 2003; Jespers et al., 2014). These observations were further buttressed by terminal restriction, fragment polymorphism and shallow profiling of the 16S rRNA ribosomal gene (Zhou et al., 2007; Zhou et al., 2010a; Zhou et al., 2011). By pyrosequencing, Zhou et al. observed a higher prevalence of *Lactobacillus* specie in Black women (33%) compared to the 7% observed in Caucasians. Furthermore, only one or two species of *Lactobacillus* were found in the few Black participant with a *Lactobacillus* profile (Zhou et al., 2010b). Similarly, Ravel et al. (2011) characterized the vaginal microbiota of 396 women by pyrosequencing of the V1 and V2 region of the 16S rRNA gene and identified a high prevalence of BVAB in African-American women (39%) compared to the lower prevalence in Asians (18%) and Caucasians (9%). The absence of *L. jensenii* vagitype and minute proportion of *L. crispatus* in African Americans was another important observation noted in their study (Ravel et al., 2011). In keeping with this, by sequencing the V1-V3 region of the 16S rRNA gene, Fettweis et al. also described the vaginal profile of Black women to be depleted of *Lactobacillus* and rich in BVAB, including *Prevotella* and *Sneathia* (Fettweis et al., 2014). It should be noticed that these studies highlighted are reports on African women living outside Africa. Results obtained from characterizing the vaginal microbiome of African women living in Africa appear to deviate from what has been observed among Africans in the diaspora. This raises important questions about the influence of geography on the vaginal microbiome. With NGS technology, a few studies have provided insight into the vaginal microbiome of women in Africa. In an 8-week longitudinal cohort study, Jespers et al. (2017) studied the vaginal microbiota of South African, Rwandan and Kenyan women and these were reported to be relatively stable and dominated by *L. iners* (75%) and *L. crispatus* (35%). Two other studies in the South African population also reported an abundance of *L. crispatus* and *L. iners*, including an heterogenous mix of CST IV microbes in the vaginal microbiota of these women in Africa (Anahtar et al., 2015; Bayigga et al., 2019).

Similarly, Lennard et al. (2017), observed a vaginal microbiota dominated by *Lactobacillus* species and some proportions of BVAB. No remarkable differences were found between the vaginal microbiome of Nigerian and Swedish women. Anukam and colleagues reported the presence of *L. gasseri*, *L. crispatus* and high proportions of *L*. *iners* in Nigerian women (Anukam et al., 2006), which is similar to the vaginal microbiome profile that had earlier been reported in Swedish women (Vasquez et al., 2002). Bacterial vaginosis-associated vaginal profile has also been reported in African women (Torrone et al., 2018; Jespers et al., 2014). By Illumina sequencing, the vaginal microbial profile of Tanzania women was characterized and found to have a significant proportion of *Prevetolla bivia* an observation made in only a small proportion of Caucasian and African-American women in North America (Hummelen et al., 2010).

Similarly, Lennard et al. (2017) reported a vaginal microbiota dominated by *Gardnerella*, *Prevotella* and *Lactobacillus* species. Furthermore, in a longitudinal study, Gossman and colleagues sequenced the V4 region of the 16S rRNA gene and reported a diverse vaginal microbiota dominated by *G. vaginalis, Prevotella, Megasphaera, Sneathia,* and *Shuttleworthia* in 58% of the study cohort. Only few subjects had a *Lactobacillus* profile dominated by *L. iners* and *L. crispatus* (Gosmann et al., 2017). The higher prevalence of BV in Black women compared to White may be explained by differences in host genetics (Ness et al., 2003; Gajer et al., 2012; Hickey et al., 2013). Besides ethnic influence and geographical consideration the vaginal microbiome of African women may also vary due to diet (Faucher et al, 2019; Tuddenham et al., 2019), innate/adaptive immunity (Jespers et al., 2017; Torcia, 2019), hormonal flunctuation (Gajer et al., 2012; Van de Wijgert et al., 2013) and other confounding factors (Koumans et al., 2007; Peipert et al., 2008). Given the inconsistency in reports from various studies on the vaginal microbiome of African women, future studies are definitely necessary.

### Vaginal microbiome of African women during pregnancy

Pregnancy represents a unique phase, characterized by a suspension of the menstrual cycle vaginal microbiome (Genc & Onderdonk, 2011). During pregnancy, the vaginal microbiome is more enriched with *Lactobacillus* than in the non-pregnant state (Romero et al., 2014; Freitas et al., 2017). Several studies have described the vaginal microbiota in pregnant women. These studies have also noted significant differences in the vaginal profile of Black and White women. African-American ethnicity increases the likelihood for having an absence of protective *Lactobacilli* which predisposes to preterm birth and other pregnancy complications (Beigi et al., 2005; Larsson et al., 2007; Klatt et al., 2010). Since no prior study has described in details the vaginal microbiome profile of African women during pregnancy in a longitudinal fashion, researchers continue to rely on results extrapolated from African women in the diaspora. Although ethnicity may have a significant influence on the vaginal microbiome, geographical variations may also be contributory. The study of Hyman et al. (2014) observed a low proportion of *Lactobacillus* in Black women who encountered preterm birth (Hyman et al., 2014). Fettweis and colleagues made a similar observation (Fettweis et al., 2019). These findings were further reinforced by the observations made in a longitudinal study of a cohort comprising of 23 White, 5 Black and 13 Asian healthy women. A large proportion of *L. jensenni* and *L. gasseri* were reported in White and Asian women but no traces of *L. gasseri and L. jensenni* were found in the vaginal samples of the Black women in this cohort (MacIntyre et al., 2015). Conversely, a study conducted in Burkina Faso which features HIV- infected pregnant women at 36–38 weeks’ gestation reported a large number of women having a *Lactobacillus*-dominant profile comprising of three distinct clusters. The first cluster comprised of *L. iners* (77%), *L. crispatus* (11%), *L. fornicalis* (3.9%), *L. gasseri* (3.2%) and *L*.*vaginalis* (0.5%). The second cluster comprised of coagulase-negative *Staphylococcus* while the third group of bacteriomes were a mixture of microbes of the CST IV type, dominated by *Gardnerella* species (Frank et al., 2012). Obviously, a *Lactobaccillus* depleted and BV-dominated vaginal profile correlates significantly with STI and HIV (Bayigga et al., 2019), yet the study of Frank et al. reported some *Lactobacillus vagitypes* in the vaginal profile of the HIV-positive pregnant women. To bridge the gap in these discrepancies, a geographically tailored approach to vaginal microbiome science is required.

### Vaginal microbiome of African women in the postpartum period

Following the changes that occur in a woman’s physiology during the postpartum, the vaginal microbiome profile is dramatically altered. During pregnancy, estrogen in maternal circulation rises (Roy & Mackay, 1962; Siiteri & MacDonald, 1966), however during the postpartum elevated level of estrogen falls dramatically due to expulsion of the placenta (Nott et al., 1976; O’Hara et al., 1991). This suggests why any estrogen driven *Lactobacillus* during pregnancy are significantly depleted postpartum (MacIntyre et al., 2015). Only few studies have successfully described the composition of the vaginal profile in postnatal women of African descent using 16S rRNA gene sequencing. These studies have observed a predominance of BVABs, *Prevotella, Anaerococcus, Streptococcus, Atopobium and Peptoniphilus* than *Lactobaccillus* in numerous postnatal women (Poretsky et al., 2014; MacIntyre et al., 2015; DiGiulio et al., 2015; Doyle et al., 2018). Notable is a study which described the vaginal microbial profile of rural Malawian women postpartum as being dominated by *Gardnerella vaginalis* (75.7%), with minute proportions of *L. crispatus and L. iners* in 30.4% of the study population (Doyle et al., 2018). The report Doyle’s group presented is similar to other observations on the postpartum microbiome in several other populations (Poretsky et al., 2014; MacIntyre et al., 2015; DiGiulio et al., 2015; Doyle et al., 2018). These observations are interesting, given that these studies featured participants from different ethnicities with variations in sample size, sample collection methods and laboratory methods. It therefore appears that the postpartum microbiome may neither be influenced by ethnicity nor geography. Till date, only the study of Doyle et al. (2018) has described the vaginal microbiota in an African population (rural Malawian women) postpartum employing 16S rRNA sequencing (Table 2). This highlights the need for further studies on the vaginal microflora during the postpartum. Another transition requiring further study is the period of restoration from the postpartum vaginal profile to the interpregnancy (normal) profile. While MacIntyre et al. (2015) focused on a mixed ethnic cohort at 6 weeks postpartum, Doyle’s group focused on a postpartum cohort one week after delivery and followed the cohort up for up to one year, yet reported no trace of *Lactobacillus* restoration (Doyle et al., 2018). Another group observed a cohort of postnatal women for one year, yet no profound vaginal *Lactobacillus* was observed (DiGiulio et al., 2015). A large longitudinal study is therefore recommended to establish the composition of the postpartum vaginal microbiome accurately and to provide more insight into how a *Lactobacillus* profile is restored after lochia regression. The vaginal microbiome composition of sub-Saharan African women is described in Table 2.

**Table 2:** Vagina microbial profiles of sub-Saharan African women.

First Author	Country	Participants description and sequencing method	Findings
Anahtar et al. (2015)	South Africa	Black women, 16S rRNA sequencing	Vaginal profile characteristically dominated by *Gardnerella vaginalis* in 45% of participants. 37% of participants had a *Lactobacillus* dominated vaginal profile. The remaining participants (18%) had vaginal profile dominated with a heterogenous mixture of several BVAB.
Borgdorff et al. (2014)	Rwanda	174 Female sex workers between (18–47) years of age. Phylogenetic microarray analysis	The vagitypes identified included *L. iners* (74%), *L. crispatus* (16%), *L. jensenii*/*L. salivarius/* other (6%), *L. gasseri*/*L. johnsonii*/other (6%), *L. vaginalis*/other (21%), *Leptotrichia* (94%), *Prevotella* (91%), *Corynebacterium* (90%) and *Gardnerella* species (82%). Other common BV-associated anaerobes found were *Atopobium* (65% of samples), *Dialister* (61%), BVAB1 (50%), *Mobiluncus* (48%), *Sneathia* (47%) and *Megasphaera* (44%), but their prevalence was low in the *Lactobacilli*-dominated clusters but approached 100% in BV-associated clusters
Gosmann et al. (2017)	South Africa	236 Black women, 16S *r* RNA sequencing on Illumina platform	Diverse vaginal microbiome characteristically dominated by *G.vaginalis*, *Prevotella*, *Megasphaera*, *Sneathia*, and BVAB1 was observed in 58% of the women.
Lennard et al. (2017)	South Africa	Black women between 16–22 years, 16S *r* RNA sequencing on Illumina platform	Vaginal communities were clustered into *L. crispatus, L. iners* and an heterogenous mixture of anaerobes. 44% were BV positive, 13% BV intermediate, and 43 were BV negative.
McClelland et al. (2018)	Eastern African (Kenya, Uganda and Tanzania) And Southern African (South Africa, Botswana and Zambia).	Participants included sex workers, HIV-serodiscordant heterosexual couples and few pregnant and postpartum women above 14 years, Deep sequencing of 16S rRNA gene	Seven taxa, *Parvimonas* species Types 1 and 2, *Gemella asaccharolytica, Mycoplasma hominis, Leptotrichia/Sneathia, Eggerthella* species Type 1, and vaginal *Megasphaera* species.

First Author	Country	Participants description and sequencing method	Findings
** Pregnancy Vaginal Microbial Profiles in African women**			
Frank et al. (2012)	Burkina Faso	HIV-1-infected pregnant women at 36–38 weeks of gestation. 16S *r* RNA pyrosequencing	Three major clusters were observed. 47% of participants had a *Lactobaccillus* dominated vagitype (30/64), *L. iners* (77%), *L. crispatus* (11%), *L. fornicalis* (3.9%), *L. gasseri* (3.2%) and *L*. *vaginalis* (0.5%). The second cluster comprised of coagulase-negative *Staphylococci* with lesser abundance of *Lactobacilli*. The third clusters observed had a mixture of genera dominated by *Gardnerella* species.
Gudza-Mugabe et al. (2020)	Zimbabwe	356 women between (15 and 35) weeks of gestation and aged between (24–35) years. 16S *r* RNA sequencing on Illumina platform	Vaginal profile characteristically dominated by BVAB. *Prevetolla colorans*, *Gemella asaccharolytica* and *Mycoplasma. hominis* associated with PTB in HIV cohort while *L. jensenni* and *L. delbrueckii* were most abundant in uninfected women that delivered preterm.

First Author	Country	Participants description and Sequencing method	Findings
**Postpartum Vaginal Microbial Profiles in African women**			
Doyle et al. (2018)	Rural Malawi	994 postnatal Black women, 16S *r* RNA sequencing on Illumina platform	Vaginal profile of 75% of participant (752/994) dominated by *Gardnerella vaginalis.* 27.1% of participants (269/994) had less abundance of *Lactobacillus.*

**Table 3:** Cutting-edge methods for bacterial identification.

Cutting edge method	Principle	Advantage	Disadvantage
Whole Genome Sequencing (WGS)	Bacteria are identified by a Chain termination principle	The entire genome is accessed Has a high-resolution for capturing genomic information Encompasses both large and small variants omitted with targeted sequencing approaches Remits large volume of data in a short time and facilitates assembly of novel genomes Capable of identifying both causative variants and variants with unknown significance (Cirulli & Goldstein, 2010; Illumina, 2020).	Requires intensive skilled labour and expertise for accurate interpretation and organization of the huge data generated (Guan et al., 2012). Sequencing cost is expensive (Illumina, 2020).
Matrix-Assisted Laser Desorption/Ionization-Time of Flight Mass Spectrometry (MALDI-TOFMS)	Bacteria are identified based on Polypeptide finger-printing	This system is reliable, simple and convenient compared to WGS Has the ability to measure and analyze complex peptide mixtures thus ideal for measuring whole bacteria cells (Barbuddhe et al., 2008; Fagerquist, Yee & Miller, 2007; Moura et al., 2008; De Bruyne et al., 2011)	Sample preparation, the cell lysis method, matrix solutions and organic solvents procedures may affect the quality and reproducibility of bacterial MALDI-TOF MS fingerprints thus compromising accurate bacteria identification (De Bruyne et al., 2011)
The Biolog OmniLog Identification System (BIOLOG)	Bacteria are identified based on oxidase and catalase biochemical activity. Requires the production of a unique biochemical fingerprint. Bacteria are identified when these biochemical fingerprints are analyzed and compared to existing database (Pires & Seldin, 1997; Hung & Annapurna, 2004)	The Biolog system is better at identifying both Gram negative and Gram-positive fermentative bacteria (Stager & Davis, 1992; Hung & Annapurna, 2004)	Protocol requires pure cultures and the subsequent growth of the bacteria and pure culture and growth which is time consuming especially slow-growing, fastidious non-culturable bacteria (Morgan et al., 2009)
Ribotyping	Bacterial are identified by ribotyping sequence differences in ribosomal RNA (rRNA) also known as Ribotyping finger printing. Ribotying involves the use of rRNA as probe to detect chromosomal restriction fragment length polymorphisms (RFLPs) (Kivanç, Vilmaz & Cakir, 2011; Inglis et al., 2002)	The Ribotyping device used determines the ribotypes of diverse bacteria isolates and permits the differentiation of molecular typing data. This comparison allows for accurate identification of several bacterial species from similar family or genus level (Inglis et al., 2002; Kivanç, Vilmaz & Cakir, 2011)	Requires intensive skilled labour and expertise for accurate interpretation and organization since several discriminating molecular typing data on all isolates requires analysis
Shotgun Sequencing	Bacteria are identified by a chain termination principle	Provide information concerning the functional relevance of gene due to its high taxonomic resolution compared to 16S sequencing (Poretsky et al., 2014; Claesson et al., 2009; Brown et al., 2019). Evaluate the viral constituents of the microbiome (viromes) (Ferretti et al., 2017).	More expensive, requires greater expertise, have a more challenging workflow and allows contaminated DNA fragment to be sequenced simultaneously with microbial DNA (Brown et al., 2019)

### Alternative platform for bacterial identification

Over a decade after the recommendations by the HMP, we have witnessed a growing body of literatures deployed the 16S rDNA sequencing for bacteria identification. Although it’s been a reliable and convenient method of bacterial species identification, it has some shortfalls. It is difficult for bacteria that share similar gene sequence to be differentiated at specie level. When sequences are aligned wrongly, bacteria species are matched incorrectly. Other pitfalls with this technique are hitches with purity of bacteria isolates and sequencing artefacts which introduce errors into a DNA database which mostly likely is interpreted as an existing or reference database for new studies thus hampering accurate bacterial identification (Tshikhudo et al., 2013). Alternative cutting-edge technologies are recommended to facilitate bacteria identification even further (Table 3).

## Conclusion

NGS applications have revealed novel frontiers in microbiome research by strikingly providing phylogenetic and functional portraits of the vaginal microbial communities, including microbes that have not yet been cultivated by traditional method. We described here the 16S rRNA gene sequencing, a commonly deployed NGS platform in deciphering the vaginal microbial communities. On the basis of published literature, vaginal microbiome studies in the African population mainly features non-pregnant healthy and diseased cohorts. Future studies should consider providing insight into the pregnancy vaginal microbiome in healthy cohorts, both in cross- sectional and longitudinal fashion. A refined longitudinal multicenter study is recommended so as to critically study the influences of personal behaviors, hygiene practices, host characteristics and other maternal covariates on the vaginal microbiome during pregnancy. The study on the postpartum vaginal microbiome identified in the African population concluded by emphasizing the need for a better understanding of the complex postpartum vaginal community profile. This therefore calls for more large-scale studies on the postpartum vaginal microbiome. The commonly deployed 16S rRNA gene sequencing has enabled the identification of the distinct vaginal bacterial communities but, with some geographical and ethnic discrepancies observed across various populations, more sophisticated high-throughput platforms are recommended to exhaustively clarify inconsistencies between existing reports. This move would offer a paradigm to both clearly decipher discrepancies in the vaginal microbiome of women of similar ethnicities in different geographical regions and also identify novel potential symbionts and pathobionts in the vagina. Ultimately, NGS approach represents a giant step forward in the direction toward individualized medicine. Important breakthroughs in the prediction of accurate treatment and therapeutic interventions, for vaginal imbalances in sub-Saharan African women is envisaged.

## Abbreviations

BV: Bacterial Vaginosis
BVAB: Bacteria associated with vaginosis
QIIME 2: Quantitative Insights into Microbial Ecology 2
PID: Pelvic inflammatory disease
ASV: Amplicon sequence variants
VMB: Vaginal microbiome
OTU: Operational Taxonomic Unit
PTB: Pre-Term Births
PPROM: Pre-Term Premature Rupture of Membranes
16S rRNA: Ribosomal profiling of the Ribosomal RNA gene
HTS: High-Throughput Sequencing
HMP: Human Microbiome Project
V: Hypervariable region
CST: Community State Types
NGS: Next-Generation Sequencing

## Additional Information and Declarations

### Data Availability

The following information was supplied regarding data availability: This is a review article, therefore there is no raw data.
